# Management of Severe Congenital Protein C Deficiency with Continuous Subcutaneous Infusion via Insulin Pump: A Pediatric Case Report

**DOI:** 10.3390/children13040515

**Published:** 2026-04-07

**Authors:** Angelo Gentile, Giordano Spacco, Nicola Minuto, Vera Morsellino, Sandro Dallorso, Angelo Claudio Molinari, Mohamad Maghnie, Marta Bassi, Laura Banov

**Affiliations:** 1 Department of Neuroscience, Rehabilitation, Ophthalmology, Genetics, Maternal and Child Health, University of Genoa, 16132 Genoa, Italyspaccogiordano@gmail.com (G.S.); mohamadmaghnie@gaslini.org (M.M.); 2 Pediatric Clinic and Endocrinology Unit, Department of Medical and Pediatric Sciences, IRCCS Istituto Giannina Gaslini, 16147 Genoa, Italy; nicolaminuto@gaslini.org; 3 Home Care Service, IRCCS Istituto Giannina Gaslini, 16147 Genoa, Italy; veramorsellino@gaslini.org (V.M.); sandrodallorso@gaslini.org (S.D.); 4 Hemostasis and Thrombosis Center, Department of Hematology and Oncology, IRCCS Istituto Giannina Gaslini, 16147 Genoa, Italy; aclaudiomolinari@gaslini.org (A.C.M.); laurabanov@gaslini.org (L.B.)

**Keywords:** severe congenital Protein C deficiency, Protein C concentrate, continuous subcutaneous infusion, insulin pump, neonatal care

## Abstract

**Highlights:**

**What are the main findings?**

- Continuous subcutaneous infusion of Protein C concentrate via an insulin pump was feasible and safe in a child with severe congenital Protein C deficiency.
- Stable Protein C activity levels were maintained with progressive reduction of the weight-adjusted daily dose compared with intermittent intravenous administration.

**What are the implications of the main findings?**

- Continuous subcutaneous infusion may represent a viable long-term management strategy in patients requiring chronic Protein C replacement.
- Use of programmable insulin pumps can facilitate home-based therapy while avoiding prolonged central venous access.

**Abstract:**

Severe congenital Protein C deficiency (SCPCD) is a rare autosomal recessive thrombophilia that typically presents in the neonatal period with early-onset life-threatening thrombotic complications. We report the case of a female infant who presented at birth with digital ischemic necrosis and laboratory evidence of consumptive coagulopathy consistent with neonatal purpura fulminans. Severe Protein C deficiency was confirmed by markedly reduced Protein C activity (<0.03 IU/mL) and compound heterozygous variants in the *PROC* gene. After initial stabilization and intermittent intravenous Protein C replacement, pharmacokinetic assessment showed marked peak–trough variability. Continuous subcutaneous infusion of Protein C concentrate was therefore initiated using a programmable insulin pump in combination with oral anticoagulation. This strategy achieved stable Protein C activity levels, allowed progressive reduction of the weight-adjusted replacement dose, and enabled removal of the central venous catheter. Continuous subcutaneous infusion of Protein C concentrate via an insulin pump, combined with oral anticoagulation, may represent a feasible long-term therapeutic option in selected patients with SCPCD.

## 1. Introduction

Protein C is a vitamin-K-dependent anticoagulant synthesized in the liver and activated on the endothelial surface. Activated Protein C (APC) downregulates thrombin generation by inactivating factors Va and VIIIa, thereby preserving microvascular patency [1].

Severe congenital Protein C deficiency (SCPCD) is a rare autosomal recessive thrombophilia caused by biallelic loss-of-function mutations in the *PROC* gene [2]. The estimated incidence of homozygous or compound heterozygous forms is approximately 1 per 4 million live births, although the true prevalence is likely underestimated due to fetal demise and early neonatal mortality [3].

SCPCD typically presents within the first 72 h of life with neonatal purpura fulminans and disseminated intravascular coagulation. Prenatal thrombosis of retinal and cerebral vessels is common and may result in blindness or severe neurological sequelae. Cerebral infarction, with or without hemorrhage, may occur even in the absence of purpura fulminans [4].

Diagnosis is primarily suspected in neonates presenting with purpura fulminans or unexplained thrombotic events. Laboratory evaluation reveals markedly reduced or undetectable plasma Protein C activity, typically <0.01 IU/mL in severe forms. Functional assays should be interpreted according to age and clinical context, and molecular analysis of the *PROC* gene confirms the diagnosis [5].

Prompt initiation of Protein C replacement therapy is essential once the diagnosis is suspected. Plasma-derived Protein C concentrate is administered intravenously in the acute phase, while subcutaneous administration, although not formally licensed, has been used off-label for long-term prophylaxis for more than two decades and is widely reported in the literature [4]. Subcutaneous delivery may reduce complications related to central venous access and is typically performed as intermittent bolus injections, administered daily or at longer intervals depending on the dosing regimen. Despite its longer half-life compared with the intravenous route, intermittent subcutaneous administration may still result in variability in plasma Protein C activity [6].

Continuous subcutaneous infusion (CSI) represents a distinct delivery strategy, aiming to provide more stable pharmacokinetic exposure and steadier Protein C levels over time. This approach can be implemented using a programmable insulin pump, a device designed to deliver continuous, adjustable infusion through a subcutaneous cannula connected to a refillable reservoir, allowing precise modulation of infusion rates over 24 h [7]. To date, only isolated reports have described the use of CSI via an insulin pump for Protein C replacement [8].

Here, we report the management of a neonate with severe congenital Protein C deficiency treated with continuous subcutaneous infusion, highlighting the clinical rationale, technical considerations, and short-term outcomes of this strategy.

## 2. Case Report

### 2.1. Initial Presentation and Diagnosis

The patient was a female infant born at 37 weeks of gestation by urgent cesarean section due to a pathological cardiotocographic trace.

At birth, necrosis of the second and third toes of the left foot was observed, raising suspicion of an underlying thrombotic disorder.

On day three of life, she was referred to a tertiary pediatric center for further evaluation. Laboratory investigations revealed thrombocytopenia, hypofibrinogenemia, and elevated D-dimer levels, consistent with disseminated intravascular coagulation. The presence of rapidly progressive digital necrosis in association with consumptive coagulopathy was considered clinically consistent with neonatal purpura fulminans. Further diagnostic work-up demonstrated markedly reduced Protein C activity (<0.03 IU/mL), confirming severe congenital Protein C deficiency (SCPCD) as the underlying cause.

Subsequently, molecular analysis confirmed compound heterozygosity for two *PROC* variants: c.718C>T (p.Arg211Trp), inherited from the father, and c.237+5G>A, inherited from the mother. Parental testing revealed reduced Protein C activity in the father (0.38 IU/mL) and borderline-low levels in the mother (0.67 IU/mL); both parents were clinically asymptomatic.

### 2.2. Initial Management

The patient initially received supportive treatment with fresh frozen plasma, fibrinogen replacement, and platelet transfusions to correct the coagulopathy. On day six of life, a central venous catheter was placed, and treatment with plasma-derived Protein C concentrate (Ceprotin^®^, Takeda Pharmaceutical, Osaka, Japan) and vitamin K was initiated.

Over the following days, reperfusion of the proximal and middle phalanges of the affected toes was observed, whereas the distal phalanges progressed to necrosis and ultimately underwent spontaneous auto-amputation.

Serial ophthalmologic examinations identified retrohyaloid hemorrhage. Brain magnetic resonance imaging (MRI) demonstrated ischemic sequelae and flow alterations without evidence of ongoing thrombosis, bilateral vitreous hemorrhage, and confirmed the presence of bilateral retinal detachment, which was subsequently treated at one month of age with vitrectomy, iridectomy, silicone and gas placement, and laser photocoagulation. Electroencephalography revealed slow-wave activity in the cortico-temporal regions.

At 3 months and 20 days of age, she was referred to our center for completion of the diagnostic work-up and further therapeutic management. Detailed laboratory trends from the initial neonatal period at the referring hospital, including D-dimer levels, were not available for review. At admission, her weight was 5.115 kg, her length was 55 cm and D-dimer levels were within the physiological range. The intravenous Protein C concentrate (Ceprotin^®^) was maintained at a dose of 100 IU/kg every 12 h via a peripherally inserted central catheter (PICC), together with vitamin K, folic acid, and iron supplementation, while rivaroxaban oral suspension (1 mg/mL) was initiated at a weight-adjusted dose of 1.6 mg three times daily (for a total daily dose of 4.8 mg).

Serial Protein C activity assays were performed. Following infusion, Protein C activity increased rapidly, reaching a peak of 1.17 IU/mL at 4 h. Levels declined to 0.85 IU/mL at 8 h and to 0.59 IU/mL at 12 h. The trough level was 0.43 IU/mL. Although trough levels remained within the therapeutic range, the observed peak–trough variability and the ongoing need for central venous access prompted consideration of long-term subcutaneous replacement therapy, as commonly adopted in patients requiring chronic management.

### 2.3. Continuous Subcutaneous Therapy and Follow-Up

However, instead of administering Protein C concentrate as intermittent subcutaneous boluses, a continuous subcutaneous infusion strategy, aimed at achieving more stable plasma Protein C activity over time, was considered. After a multidisciplinary discussion, the diabetology team was consulted to assess the feasibility of delivering Protein C concentrate via an insulin pump.

After one week, continuous subcutaneous infusion was initiated using Ceprotin^®^ delivered via a MiniMed 780G insulin pump (Medtronic^®^, Northridge, CA, USA). Ceprotin^®^ (500 IU) was reconstituted immediately prior to use and diluted in 3 mL (instead of 5 mL) to match the reservoir capacity of the pump.

The pump basal rate was set at 35 U/h, corresponding to the maximum programmable insulin delivery rate (based on a standard insulin concentration of 100 U/mL, i.e., 0.01 mL per unit). This setting resulted in an actual infusion rate of 0.35 mL/h. Given the Ceprotin^®^ concentration (500 IU in 3 mL), this corresponded to an effective delivery of approximately 58 IU/h, allowing administration of the full 500 IU dose over approximately 8.5 h.

**Figure 1:**
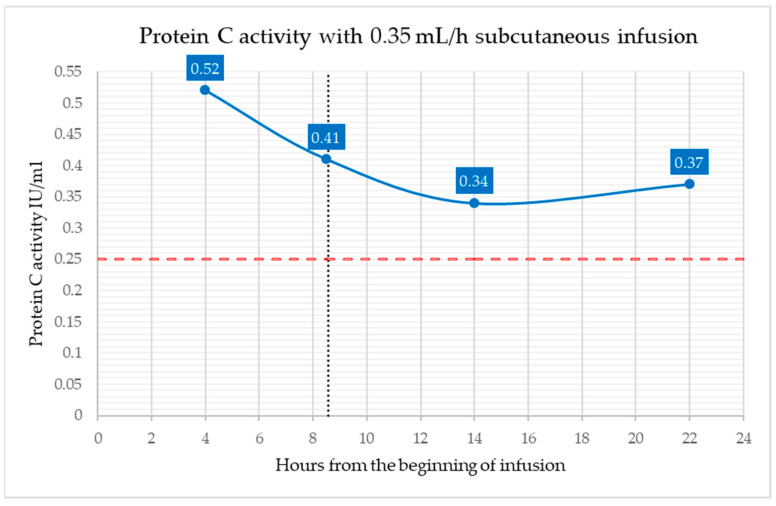
Protein C activity with 0.35 mL/h continuous subcutaneous infusion of Ceprotin^®^ over approximately 8.5 h (100 IU/Kg/day). The black dotted line indicates the end of the infusion, while the red dashed line indicates the target plasma Protein C activity (0.25 IU/mL).

Protein C activity assays were performed at multiple time points following initiation of subcutaneous infusion (after 4, 8.5, 14, and 22 h). The lowest value was observed at 14 h (0.34 IU/mL), followed by a slight increase to 0.37 IU/mL at 22 h. Therefore, as shown in Figure 1, Protein C activity levels were consistently above the therapeutic threshold of 0.25 IU/mL, confirming the stability of Protein C activity during and following the infusion.

The result was considered satisfactory, and the following day another subcutaneous infusion was performed. Twenty-two hours after the beginning of the second subcutaneous infusion, a baseline level of Protein C activity of 0.36 IU/mL was recorded.

**Figure 2:**
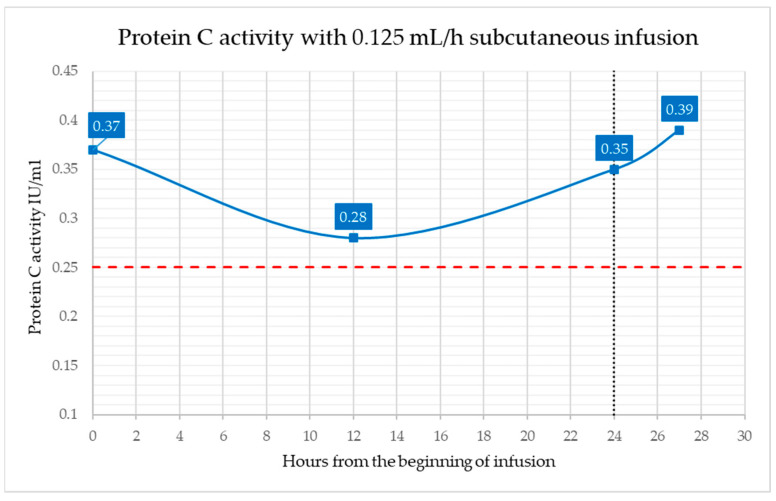
Protein C activity with 0.125 mL/h continuous subcutaneous infusion of Ceprotin^®^ over approximately 24 h (100 IU/Kg/day). The black dotted line indicates the end of the infusion, while the red dashed line indicates the target plasma Protein C activity (0.25 IU/mL).

One week later, the infusion protocol was modified. The basal rate was reduced to 12.5 U/h, corresponding to an infusion rate of 0.125 mL/h. Given the Ceprotin^®^ concentration (500 IU in 3 mL), this resulted in an effective delivery of approximately 21 IU/h, allowing administration of the full 500 IU dose over 24 h. To facilitate practical management, a brief interruption of approximately 3 h between infusion cycles was introduced. Protein C activity was measured at 12 and 24 h from the start of infusion, as well as 3 h after temporary suspension. As shown in Figure 2, Protein C activity levels were consistently maintained above the threshold with the new regimen, indicating its efficacy and reliability.

Caregiver training on pump management and infusion set handling was initiated during hospitalization. The parents received supervised instruction on pump handling, drug reconstitution, daily reservoir refill, and recognition of potential complications, including pump malfunction, cannula occlusion or displacement. Once adequate competence and autonomy had been achieved, the patient was discharged, and continuous subcutaneous Protein C replacement therapy was continued at home. Initially, the parents were supported by the institutional Home Care Service team and gradually became fully autonomous in managing both the pump and the infusion set. A structured program of periodic re-education and clinical follow-up was implemented, with regular reassessment of caregiver competence by both hospital and home care teams. Caregivers were instructed on appropriate drug storage, including precautions to avoid heat exposure and maintain a controlled ambient temperature. As the treatment period occurred during cooler months, specific guidance for drug handling during warmer conditions will be addressed subsequently.

For safety reasons, the central venous catheter was temporarily maintained while the stability of Protein C activity levels and the parents’ autonomy in pump management were being assessed.

After the patient reached 6 kg in weight, Protein C activity was re-evaluated. Protein C activity levels resulted consistently above 0.25 IU/mL with a daily dose of 500 IU of Ceprotin^®^ (approximately 80 IU/kg/day).

At 5 months and 24 days of age, given the stability of Protein C activity levels, replacement therapy was successfully de-escalated to 500 IU of Ceprotin^®^ (approximately 80 IU/kg) administered every 48 h, delivered by continuous subcutaneous infusion over approximately 20 h. The target plasma Protein C activity range was defined between 0.2 and 0.3 IU/mL. This regimen proved effective and stable, with Protein C activity levels consistently remaining within the target range.

At 6 months and 12 days of age, given the stability of Protein C activity levels under continuous subcutaneous infusion and the autonomy achieved by the parents in pump management, the central venous catheter was removed. During the same hospitalization, endogenous Protein C production was assessed by measuring Protein C activity for 48 h after suspension of Ceprotin^®^ subcutaneous infusion. A progressive decrease in Protein C activity was observed, reaching 0.13 IU/mL at 48 h. These findings indicated insufficient endogenous Protein C production, and replacement therapy was therefore continued.

At the time of writing, the patient is 9 months old. She is growing regularly and has reached a body weight of 7.5 kg. Treatment with Ceprotin^®^ is currently administered every 48 h via the insulin pump (500 IU per infusion cycle, delivered at an infusion rate of 0.15 mL/h) together with a weight-adjusted dose of rivaroxaban.

Since the initiation of continuous subcutaneous infusion of Ceprotin^®^ in combination with rivaroxaban, no recurrence of purpura fulminans or other cutaneous signs of breakthrough coagulopathy has been observed. Over five months of follow-up, the patient has remained clinically stable, with no thrombotic or infectious complications and no laboratory evidence of subclinical coagulopathy.

Given that Ceprotin^®^ is a plasma-derived product, regular serological screening for blood-borne pathogens was performed during follow-up and remained negative. In light of the limited long-term experience with this condition, a protocol for intensified surveillance by the home care team during potential high-risk periods, such as infections and vaccinations, was implemented. To date, no breakthrough thrombotic events or clinical complications have occurred during these trigger situations, and the patient successfully completed the scheduled pediatric vaccination program without adverse sequelae.

## 3. Discussion

Severe congenital Protein C deficiency (SCPCD) is a rare but life-threatening thrombophilia that often presents in the neonatal period with purpura fulminans and disseminated intravascular coagulation. Prompt initiation of Protein C replacement therapy is essential once the diagnosis is suspected. Plasma-derived Protein C concentrates remain the cornerstone of treatment, with Ceprotin^®^ and Protexel^®^ (LFB Biomedicaments, Les Ulis, France) being the most widely available formulations. Intravenous administration is generally recommended in the acute phase, while subcutaneous administration is often preferred for long-term prophylaxis. Current recommendations suggest maintaining plasma Protein C activity levels above 0.5 IU/mL during the initial phase of treatment, with levels above 0.25 IU/mL considered adequate once clinical stabilization is achieved [4].

Despite the central role of Protein C replacement therapy, the long-term management of SCPCD remains challenging. Ceprotin^®^, the most widely used Protein C concentrate, is currently approved only for intravenous administration, although subcutaneous administration is commonly used in clinical practice for chronic replacement therapy [4]. Evidence-based recommendations regarding optimal dosing regimens are lacking, and reported subcutaneous administration strategies vary widely across published case reports and small case series [9]. Similarly, the optimal anticoagulation strategy in patients with SCPCD remains uncertain. Only a few cases have been described of long-term management of SCPCD with direct oral anticoagulants (DOACs) alone [10,11] or in combination with activated Protein C concentrate [12]. In particular, the association with VKAs has been reported to allow a reduction in both anticoagulant and Protein C replacement dosage in some cases [4].

In the present report, we describe the management of an infant with SCPCD treated with continuous subcutaneous infusion of Ceprotin^®^ using an insulin pump in combination with rivaroxaban. Both the Protein C concentrate and the insulin pump were used off-label. To the best of our knowledge, this represents the second reported case in the literature of Protein C concentrate administered via an insulin pump.

In the case reported by Piccini et al. [8], continuous subcutaneous infusion of Protein C using an insulin pump was introduced early in life, approximately 30 days after birth, primarily to overcome difficulties in maintaining venous access. In that report, the insulin pump was used as a temporary strategy to bridge the transition toward anticoagulant therapy. In contrast, in our patient, the insulin pump was used as part of a structured long-term therapeutic strategy aimed at maintaining stable Protein C activity levels while enabling home-based management and avoiding prolonged use of central venous access. In both cases, no adverse events related to pump administration were reported.

In our experience, the insulin pump proved to be a reliable method for delivering continuous subcutaneous Protein C replacement therapy. Across all attempted infusion regimens, Protein C activity levels remained consistently above the therapeutic threshold of 0.25 IU/mL. Notably, the stability achieved with continuous infusion allowed progressive de-escalation of the weight-adjusted Protein C dosage while maintaining adequate activity levels. The infusion regimen consisting of subcutaneous administration every other day proved to be effective and stable, while also reducing the overall required amount of Protein C concentrate.

An additional advantage of this strategy was the possibility of training the parents to independently manage pump therapy at home. The user-friendly nature of the insulin pump facilitated caregiver education and allowed safe continuation of therapy outside the hospital setting. Moreover, the use of continuous subcutaneous infusion ultimately enabled the removal of the central venous catheter, thereby reducing the risk of catheter-related infectious and thrombotic complications, which are well-recognized concerns in patients requiring long-term intravenous therapy [13].

However, the use of an insulin pump for Protein C administration also presents some limitations. In particular, the device used in this case has a fixed reservoir capacity of 3 mL and a maximum programmable infusion rate of 0.35 mL/h, which led us to successfully reduce the final volume of the Protein C concentrate. While these limitations did not affect drug delivery in our patient due to the relatively low protein C requirements during infancy, they could represent a constraint in older children requiring higher replacement doses. In such cases, the insulin pump may still represent a temporary solution, for example, while waiting for the availability of an alternative infusion device or while attempting to discontinue central venous access. In these cases, a second 3 mL reservoir could be inserted to prolong the administration and increase the total dose of concentrate.

An important consideration in our approach is the biochemical stability of Protein C concentrate when held at body temperature in the insulin pump reservoir for extended periods. While the FDA label for Ceprotin^®^ recommends administration within 3 h of reconstitution, and no data specifically validate its stability in pump reservoirs over 20–24 h, the maintenance of therapeutic Protein C activity levels in our patient provides functional evidence of adequate stability under these conditions [14]. Indirect evidence from related proteins support this observation. Plasma-derived coagulation factor concentrates (Factor VIII and Factor IX) have shown retention of >90–95% activity for up to 48–168 h at 37 °C in pump systems [15,16,17,18]. Insulin pump studies have also shown preserved protein integrity with insulin aspart under similar conditions despite continuous mechanical stress [19]. The intrinsic thermal stability of Protein C (melting temperature ~62 °C) further supports the plausibility of structural preservation at body temperature. In addition, Ceprotin^®^ contains stabilizing excipients (human albumin, trisodium citrate), which may enhance stability [14].

Given the long-term use of a plasma-derived product and despite the robust viral inactivation steps that have characterized the modern safety profile of Protein C concentrates, a formal safety monitoring protocol was implemented in our case. This included semiannual serological screening for blood-borne pathogens and, to date, no viral transmissions or associated adverse events have occurred, consistent with current real-world registry data.

The development of neutralizing anti-Protein-C antibodies remains a theoretical concern in patients receiving long-term replacement therapy. However, no cases of inhibitor formation have been reported in clinical trials or large international registries, even among patients with severe congenital deficiency and high cumulative exposure. Consequently, routine antibody testing is not currently recommended and was not performed in our patient, who maintained a stable clinical response throughout follow-up [20].

Regarding laboratory monitoring, D-dimer was not routinely measured as a marker of ongoing coagulopathy or treatment efficacy. While it is a well-established diagnostic tool for excluding venous thromboembolism in adults, its role in monitoring anticoagulation, particularly in pediatric patients receiving both Protein C concentrate and rivaroxaban, remains unproven. In addition, current pediatric evidence suggests limited sensitivity and specificity, as levels are frequently influenced by infections or inflammatory states, potentially leading to misleading interpretations [18].

Another relevant aspect of our therapeutic strategy is the potential synergy between continuous subcutaneous Protein C infusion and concomitant rivaroxaban. Rivaroxaban is a direct Factor Xa inhibitor that reduces thrombin generation without affecting Protein C activity or chromogenic Protein C assays [21,22]. Therefore, the therapeutic Protein C activity levels observed in our patient can be attributed to the continuous subcutaneous infusion rather than to any pharmacologic effect of rivaroxaban. The combination of these agents provides complementary antithrombotic mechanisms, with Protein C replacement restoring the natural anticoagulant pathway and rivaroxaban reducing thrombin generation through Factor Xa inhibition [21,23]. This synergy may have contributed to the efficacy observed in our patient, potentially allowing for lower Protein C doses and improving the overall risk–benefit profile. Notably, rivaroxaban offers a theoretical advantage over vitamin K antagonists (VKAs) in this setting. VKAs reduce endogenous Protein C synthesis and may induce a transient hypercoagulable state during initiation, requiring careful Protein C replacement to prevent warfarin-induced skin necrosis [24]. In contrast, rivaroxaban does not affect Protein C production, potentially simplifying management [25]. This is, to our knowledge, the only reported case of long-term Protein C replacement via an insulin pump. However, several limitations should be acknowledged. First, this report is based on a single patient, limiting the generalizability of the findings. In addition, both the use of plasma-derived Protein C concentrate via continuous subcutaneous infusion and its delivery through an insulin pump are off-label. Furthermore, data on long-term safety, pharmacokinetics, and clinical outcomes are lacking, and no studies have specifically evaluated the stability of Protein C concentrate in insulin pump reservoirs at body temperature over extended periods. Although our clinical and biochemical findings support the feasibility of this approach, formal stability studies, including assessment of Protein C activity, antigen levels, and potential aggregation or degradation over time, as well as prospective pharmacokinetic analyses, are needed. These limitations highlight an important knowledge gap that should be addressed through controlled studies before widespread adoption of this technique.

Overall, our experience suggests that continuous subcutaneous infusion of Protein C using an insulin pump may represent a feasible therapeutic option in selected patients with SCPCD, particularly during early childhood when dosing requirements are compatible with the technical characteristics of the device. Further studies are needed to better define optimal dosing regimens and the role of concomitant anticoagulation in this setting.

## 4. Conclusions

This case report suggests that continuous subcutaneous infusion of Protein C using an insulin pump, in combination with oral anticoagulation, may represent a feasible, safe, and effective strategy for the long-term management of severe congenital Protein C deficiency. In our patient, this approach allowed stable Protein C activity levels, facilitated home-based therapy, and enabled removal of the central venous catheter, thereby reducing the risk of catheter-related complications. Importantly, the achievement of therapeutic Protein C activity levels provides functional evidence that the concentrate maintained adequate biochemical stability in the insulin pump reservoir at body temperature, consistent with stability data reported for other coagulation factor concentrates under similar conditions. Nevertheless, formal in vitro stability studies and prospective pharmacokinetic analyses are warranted to validate this delivery method before widespread clinical adoption. This experience also highlights the importance of a multidisciplinary approach in managing complex and rare conditions such as severe congenital Protein C deficiency.

## Data Availability

The original contributions presented in this study are included in the article. Further inquiries can be directed to the corresponding author.

## Abbreviations

The following abbreviations are used in this manuscript:

SCPCD	Severe Congenital Protein C Deficiency
APC	Activated Protein C
CSI	Continuous Subcutaneous Infusion
MRI	Magnetic Resonance Imaging
PICC	Peripherally inserted central catheter
DOACs	Direct Oral Anticoagulants
VKAs	Vitamin K Antagonists
